# Validation of smartwatch electrocardiogram intervals in children compared to standard 12 lead electrocardiograms

**DOI:** 10.1007/s00431-024-05648-7

**Published:** 2024-06-25

**Authors:** Julia Ernstsson, Birgitta Svensson, Petru Liuba, Constance G. Weismann

**Affiliations:** 1 Department of Clinical Sciences Lund, Lund University, Lund, Sweden; 2 Department of Pediatric Cardiology, Pediatric Heart Center, Skåne University Hospital, Lund, Sweden; 3 Department of Pediatric Cardiology and Pediatric Intensive Care, Ludwig Maximilium University, Munich, Germany

**Keywords:** Smartwatch, Electrocardiogram intervals, QT, Congenital heart disease, Pediatric cardiology, Telemonitoring

## Abstract

Lay people are now able to obtain one-lead electrocardiograms (ECG) using smartwatches, which facilitates documentation of arrhythmias. The accuracy of smartwatch derived ECG intervals has not been validated in children though. Home-based monitoring of ECG intervals using a smartwatch could improve monitoring of children, e.g. when taking QTc prolonging medications. The aim of this study was to validate the ECG intervals measured by smartwatch in comparison to standard 12-lead ECGs in children and adolescents. Prospective study of children (age 5—17 years) at the outpatient clinic of a national pediatric heart center. Patients underwent a smartwatch ECG (ScanWatch, Withings) and a simultaneous standard 12-lead ECG. ECG intervals were measured both automatically and manually from the smartwatch ECG and the 12-lead ECG. Intraclass correlation coefficients and Bland–Altman plots were performed. 100 patients (54% male, median age 12.9 (IQR 8.7–15.6) were enrolled. The ICC calculated from the automated smartwatch and automated 12-lead ECG were excellent for heart rate (ICC 0.97, *p* < 0.001), good for the PR and QT intervals (ICC 0.86 and 0.8, *p* < 0.001), and moderate for the QRS duration and QTc interval (ICC 0.7 and 0.53, *p* < 0.001). When using manual measurements for the smartwatch ECG, validity was improved for the PR interval (ICC 0.93, *p* < 0.001), QRS duration (ICC 0.92, *p* < 0.001), QT (ICC 0.95, *p* < 0.001) and QTc interval (ICC 0.84, *p* < 0.001).

*Conclusion*: Automated smartwatch intervals are most reliable measuring the heart rate. The automated smartwatch QTc intervals are less reliable, but this may be improved by manual measurements.

**What is Known:**

- *In adults, smartwatch derived ECG intervals measured manually have previously been shown to be accurate, though agreement for automated QTc may be fair.*

**What is New:**

- *In children, automated smartwatch QTc intervals are less reliable than RR, PR, QRS and uncorrected QT interval.*

- *Accuracy of the QTc can be improved by peroforming manual measurements.*

## Introduction

Smartwatches have become increasingly popular for monitoring heart rate (HR), oxygen saturation, sleeping patterns and electrocardiograms (ECG). The one-lead smartwatch ECG is easily performed at home by lay people compared to a standard 12-lead ECG. Smartwatch ECGs are used to monitor e.g. for atrial fibrillation and some devices have both certification by the United States Food and Drug Administration (FDA) and Conformité Européenne (CE) for this use [1, 2]. Other potential applications may be monitoring patients with suspected other arrhythmias like supraventricular or ventricular tachycardias as ECG tracings may be shared with the medical provider. Some smartwatches also provide ECG intervals such as the QTc. This may be particularly relevant for patients taking QTc prolonging medications or those with long QT syndrome. Currently, these children and adolescents undergo sporadic ECG testing, which does not take into account day-to-day variability. If QTc measured automatically by smartwatch is valid, this may provide a unique opportunity for long-term monitoring of these patients.

Several studies have been performed on adults with and without cardiac diseases comparing a standard 12-lead ECG to smartwatch ECGs. Some have compared different leads of the12-lead ECG to a corresponding smartwatch ECG recording (i.e. leads I, II, III, V1-6) and found good agreement as well as high sensitivity and specificity even for detecting ST-segment changes in patients with acute coronary syndrome [3, 4]. For adults, good agreement between 12-lead and smartwatch ECG has been described for the QTc interval as well [5].

Pediatric smartwatch data is rather limited. One study has shown an 84% sensitivity and 100% specificity for detecting abnormalities by smartwatch which were present on the 12-lead ECG [6]. Others have evaluated the diagnostic accuracy of manually measured ECG intervals by the Alivecor Kardia mobile device (mHealth) or the Apple Watch 6 compared to 12-lead ECGs in children and found overall good accuracy [7, 8].

No study has previously been done validating the ECG intervals measured by smartwatch automatically and manually in children and adolescents. The aim of this study was to compare the ECG intervals of a smartwatch with that of a standard 12-lead ECG in children and adolescents at a pediatric heart center. We hypothesized that automated and manual measurements of ECG intervals are valid in children when using 12-lead ECGs as a reference. Validating the smartwatch´s accuracy is an important step towards frequent home-based ECG-monitoring for children taking for example QTc prolonging drugs.

## Material and methods

This is a prospective study performed in the outpatient clinic of the pediatric heart center at Skåne University Hospital in Lund, Sweden, between September and October 2023. Patients aged 5–17 years who came for a routine outpatient pediatric cardiology visit were asked to participate in the study. Exclusion criteria were inability to cooperate or provide informed consent and/or assent e.g. due to developmental disability. Patient demographics, such as length, weight and prior cardiac interventions were collected at the time of the visit.

**Fig. 1:**
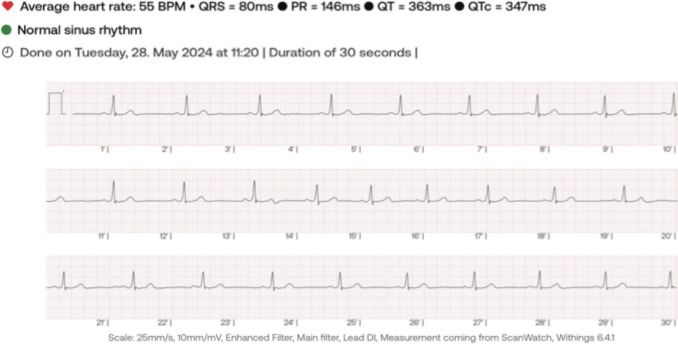
Example of a ScanWatch ECG tracing output

A smartwatch ECG and a standard 12-lead ECG were recorded simultaneously, according to the manufacturer’s instructions. The smartwatch ECG was obtained using a sinlge commercially available smartwatch, Withings ScanWatch (first generation, version 6.3.4, Withings SA, Issy les Moulineaux, France) at a pre-set paperspeed of 25 mm/s. It consists of a single peripheral lead, equivalent to lead I, created from a circuit between an electrode at the back of the watch in contact with the skin and the contralateral finger resting on the watch dial, which also functions as an electrode (Fig. 1).

A standard 12-lead ECG, EC Sense Resting ECG System (Cardiolex Medical AB, Solna, Sweden) was used as the gold standard at a standard recording speed of 50 mm/s. For each method, both automated and manual measurements were documented for HR/RR-interval, PR interval, QRS duration, QT interval and QTc interval. The median of three beats was used. The PR interval, QRS duration and QT intervals were measured on the wave following the RR interval. The QTc interval (unit: milliseconds) was determined using Bazett´s formula; QT-interval / (preceding RR interval^2) [9]. On the 12-lead ECG all manual measurements were done in both lead I and II, since lead II is the standard lead used for manual measurements on the 12-lead ECG, but the smartwatch collects information corresponding to lead I.

### Statistical analysis

Study data were collected and managed using REDCap (Research Electronic Data Capture) hosted at Lund University [10, 11]. ECG times and intervals were compared between 12-lead ECG (automatic, manual lead I and manual lead II) as well as smartwatch (automatic and manual) measurements. As the data were not normally distributed, continuous variables data are presented as median (inter-quartile range, IQR). Differences between groups were assessed using Friedman’s test for related samples. Intraclass correlation coefficients (ICC) with 95% confidence intervals (95%CI) and mean difference (95% CI) between the smartwatch and 12-lead ECG-measurements were performed for each parameter. An ICC ≥ 0.9 was defined as excellent, 0.75–0.89 as good, 0.5–0.74 as moderate and < 0.5 a poor validity [12]. Bland–Altman plots were done showing the difference between the automated readings by smartwatch vs 12-lead ECG for each parameter. Differences > 30 ms between the smartwatch and 12-lead ECG measurements were arbitrarily considered clinically relevant outliers. Outliers were checked manually and the likely reason was determined individually (i.e. automatically determined interval was matched to the ECG tracing manually in order to determine the likely source of error).

All measurements were obtained by a single observer (J.E.). Interobserver variability was assessed using ICC for 15 smartwatch ECGs by a second experienced observer (C.G.W.) who was blinded to the measurements of the main observer. Statistical tests were performed using Statistical Package for Social Sciences, version 28 (IBM SPSS, Chicago, IL). The Swedish Ethical Review Authority has approved this study in accordance with the Declaration of Helsinki (#2023–02009). Written informed consent were obtained for all participants.

## Results

### Demographics

In the study, 100 consecutive patients between age 5 and 17 at the pediatric heart center of Skåne University Hospital Lund, Sweden, were enrolled. Sixteen patients were considered for the study but did not participate due to comorbidity such as severe autism or Down syndrome, or because they chose not to participate. The median age was 12.9 (IQR 8.7–15.6) years, and 54 (54%) participants were male (Table 1).

**Table 1:** Study characteristics of the study participants

Variable	*N*	Cohort characteristics
Age [years], median (IQR)	100	12.9 (8.7–15.6)
Male, *n* (%)	100	54 (54%)
Length [centimeters], median (IQR)	98	155.8 (128.4–170.2)
Weight [kilogram], median (IQR)	99	46.4 (30.0–63.7)
First visit, *n* (%)	100	24 (24%)
Prior cardiac surgery, *n* (%)	98	36 (36%)
Prior catheter intervention, *n* (%)	95	12 (13%)

Data presented as median (interquartile range, IQR) or number, *n* (%)

**Fig. 2:**
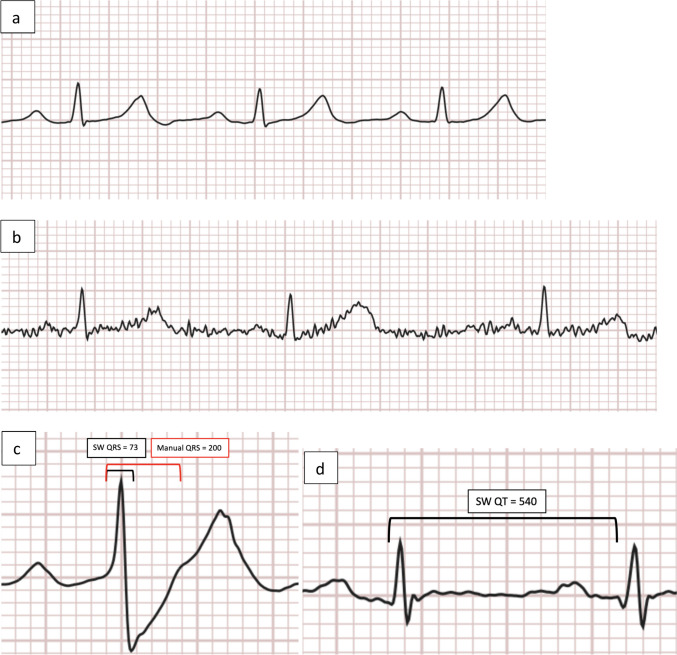
Examples of smartwatch ECG tracings with; **a** good quality. **b** low quality due to baseline artefacts. **c** mismatch between the automated (73 ms) and manual (200 ms) measurement of the QRS duration. **d** a flat T-wave where an automated measurement was performed but a manual measurement was deemed not feasible. ECG = Electrocardiogram. Ms = Milliseconds. QRS = QRS duration. QT = QT interval. SW = Smartwatch

In some cases, it was not possible to perform certain measurements (e.g. PR interval in complete AV block or isoelectric T wave; Fig. 2a-b). Manual smartwatch measurements could be performed on more ECGs than automated measurements (Table 2).

**Table 2:** Number of collected measurements from the 12-lead ECG and the smartwatch. Automated measurements from the 12-lead ECG and smartwatch ECG as well as manual measurements from the 12-lead ECG´s lead I and II and the smartwatch

	12-lead ECG			Smartwatch ECG	
	Automated	Manual lead I	Manual lead II	Automated	Manual
HR	100	100	100	98	100
RR	100	100	100	98	100
PR	96	91	93	90	94
QRS	100	100	100	97	100
QT	100	96	98	85	95
QTc	100	96	98	85	95

*ECG* Electrocardiogram; *HR* Heart rate; *PR* PR interval; *QRS* QRS duration; *QT* QT interval; *QTc* QTc interval; *RR* RR interval.

Data presented as number of measurements

Overall, manual and automated measurements of the 12-lead and smartwatch ECGs differed significantly for all variables (Table 3).

**Table 3:** Electrocardiogram median (interquartile range) durations in milliseconds

	12-lead ECG			Smartwatch ECG		*p*-value
	Automated median (IQR)	Manual lead I, median (IQR)	Manual lead II median (IQR)	Automated median (IQR)	Manual median (IQR)	
HR	74 (68 – 85)	75 (67 – 88)	75 (67 – 88)	76 (68 – 89)	75 (65 – 87)	0.003*
RR	816 (706 – 879)	800 (685 – 900)	800 (685 – 900)	789 (678 – 879)	800 (690 – 920)	0.003*
PR	144 (126– 158)	140 (120 – 160)	140 (120 – 160)	136 (123 – 161)	140 (120 – 160)	< 0.001*
QRS	89 (82 – 102)	80 (80 – 100)	80 (80 – 100)	70 (58 – 88)	80 (80 – 100)	< 0.001*
QT	378 (350 – 408)	360 (340–400)	370 (340 – 400)	343 (323 – 370)	360 (340 – 400)	< 0.001*
QTc	424 (409 – 438)	413 (392 – 428)	415 (402 – 437)	391 (371- 412)	413 (400 – 431)	< 0.001*

*ECG* Electrocardiogram; *HR* Heart rate; *IQR* Interquartile range; *PR* PR interval; *QRS* QRS duration; *QT* QT interval; *QTc* QTc interval; *RR* RR interval. * = significant

Nonparametric Friedman test

### Level of agreement between the 12-lead and smartwatch ECG

Looking at the agreement between the different ECGs, we first focused on the agreement between the automated and manual lead I and II measurements of the 12-lead ECG. Agreement between automated and manual measurements were excellent for all parameters on the 12-lead ECG (ICC ≥ 0.9, *p* < 0.001; Table 4). Similarly, agreement between automated and manual measurements derived by smartwatch was excellent for HR, RR interval, PR interval and QT interval (ICC ≥ 0.9, *p* < 0.001; Table 4). By contrast, agreement for the QRS duration and QTc interval was worse – though still good (ICC 0.75–0.79, *p* < 0.001; Table 4).

**Table 4:** Intraclass correlation coefficient (95% confidence interval) for the 12-lead ECG and the smartwatch ECG

	12-lead automated		Smartwatch automated
	12-lead manual, lead I	12-lead manual, lead II	Smartwatch manual
HR	0.97 (0.95–0.98)*	0.97 (0.95–0.98)*	0.99 (0.99–1.00)*
RR	0.96 (0.95–0.98)*	0.96 (0.95–0.98)*	0.99 (0.99–1.00)*
PR	0.95 (0.92–0.97)*	0.95 (0.93–0.97)*	0.91 (0.86–0.94)*
QRS	0.95 (0.92–0.96)*	0.95 (0.93–0.97)*	0.75 (0.62–0.83)*
QT	0.95 (0.92–0.96)*	0.97 (0.96–0.98)*	0.92 (0.88–0.95)*
QTc	0.9 (0.84–0.93)*	0.93 (0.89–0.95)*	0.79 (0.67–0.86)*

*HR* Heart rate; *PR* PR interval; *QRS* QRS duration; *QT* QT interval; *QTc* QTc interval; *RR* RR interval. * = *p* < 0.001

Next, we compared the automated and manual smartwatch measurements to the 12-lead ECG measurements (automated and manual lead I and II; Table 5). The ICCs for the standard 12-lead ECG compared to the smartwatch ECG were all highly significant (*p* < 0.001). The ICC for the automated smartwatch and 12-lead measurements were excellent for HR (ICC ≥ 0.9, *p* < 0.001), good for the PR interval and the QT interval (ICC 0.75–0.89, *p* < 0.001), and moderate for the QRS duration and the QTc interval (ICC 0.5–0.74, *p* < 0.001). In comparison to the 12-lead ECGs, the agreement of the manual smartwatch measurements was superior to the automated smartwatch measurements for the PR interval, QRS duration, QT interval and QTc interval. The agreement for manual smartwatch measurements compared to 12-lead ECGs was good or better for all variables (ICC > 0.84).

**Table 5:** Intraclass correlation coefficient (95% confidence interval) for the automated and manual smartwatch ECG corresponding to the 12-lead ECG automatic, manual lead I and manual lead II measurements

12-lead	Smartwatch automated			Smartwatch manual		
	Automated	Manual lead I	Manual lead II	Automated	Manual lead I	Manual lead II
HR	0.97 (0.96–0.98)*	0.99 (0.98–0.99)*	0.99 (0.98–0.99)*	0.97 (0.96–0.98)*	0.98 (0.97–0.99)*	0.98 (0.97–0.99)*
PR	0.86 (0.78–0.91)*	0.86 (0.78–0.91)*	0.89 (0.83–0.93)*	0.93 (0.89–0.95)*	0.93 (0.90–0.95)*	0.92 (0.88–0.95)*
QRS	0.70 (0.55–0.80)*	0.70 (0.55–0.80)*	0.71 (0.56–0.80)*	0.92 (0.88–0.95)*	0.95 (0.92–0.96)*	0.86 (0.80–0.91)*
QT	0.80 (0.69–0.87)*	0.89 (0.84–0.93)*	0.81 (0.70–0.88)*	0.95 (0.92–0.96)*	0.96 (0.93–0.97)*	0.95 (0.92–0.97)*
QTc	0.53 (0.28–0.70)*	0.65 (0.45–0.77)*	0.52 (0.26–0.69)*	0.84 (0.76–0.89)*	0.88 (0.81–0.92)*	0.87 (0.80–0.91)*

*ECG* Electrocardiogram; *HR* Heart rate; *PR* PR interval; *QRS* QRS duration; *QT* QT interval; *QTc* QTc interval. * = *p* < 0.001

### Visualizing the agreement between the 12-lead ECG and smartwatch ECG

**Fig. 3:**
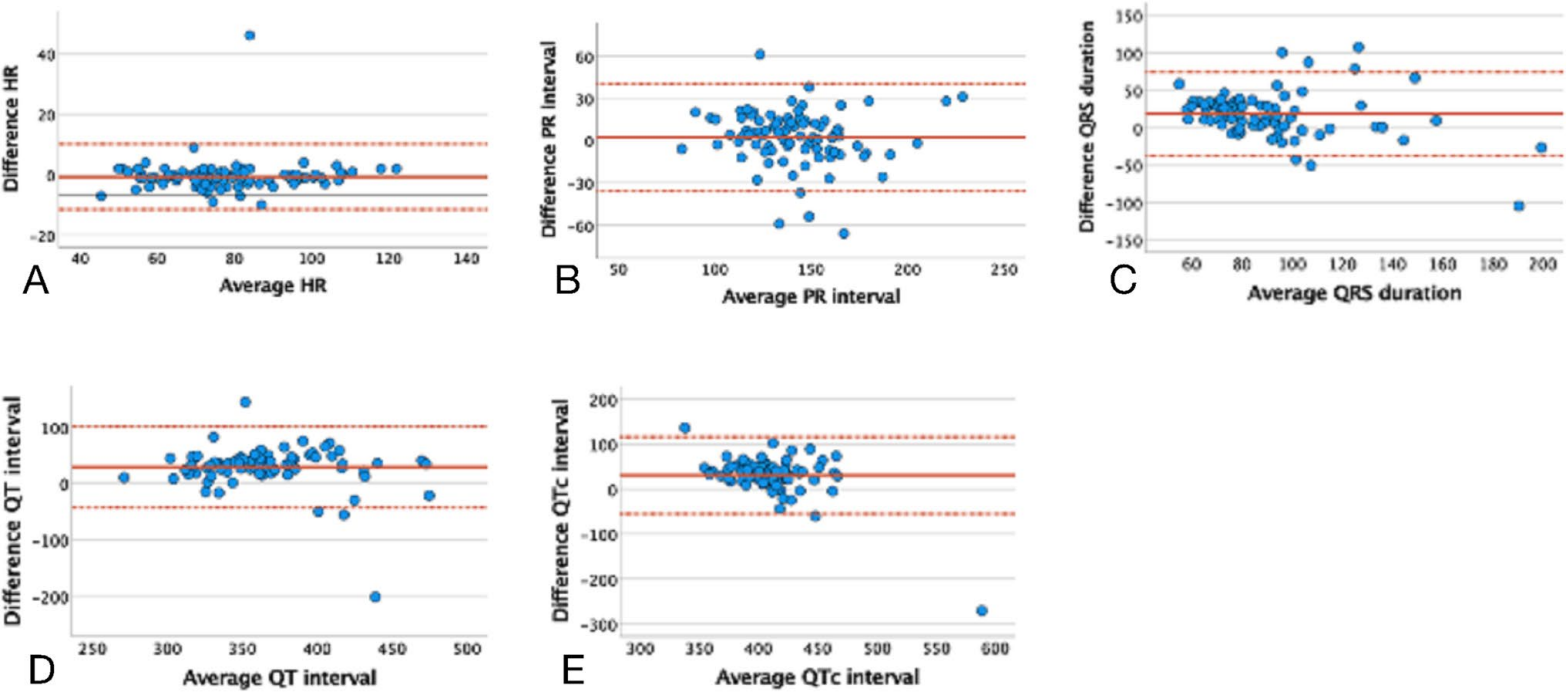
Bland Altman plot picturing the level of agreement measured automatically with a smartwatch and a standard 12-lead ECG (12-lead ECG – Smartwatch ECG). The solid red line represents the bias and the dotted lines the upper and lower limits of agreement. **a** HR (bpm). **b** PR interval (ms). **c** QRS duration (ms). **d** QT interval (ms). **e** QTc interval (ms). Bpm = Beats per minute. ECG = Electrocardiogram. HR = Heart rate. LoA = Limits of agreement. Ms = Milliseconds

In order to visualize the agreement between the automated 12-lead and smartwatch ECG, we performed Bland Altman plots for each parameter (Fig. 3a-e). There was no significant difference in HR between the two modalities (bias − 0.6 beats per minute (bpm) (95% limits of agreement (LoA) − 11.3 to + 10.2 bpm), *p* = 0.499; Fig. 3a). Only one patient (1%) was considered an outlier, with a difference of 46 bpm between the two modalities. The difference was attributed to the 12-lead ECG overestimating the HR in a patient with complete AV-block, while the smartwatch derived HR was correct.

For the PR interval, we also found no significant bias (bias 2.2 ms (95% LoA -35.8 to + 40.2), *p* = 0.403; Fig. 3b). Seven patients (8%) had a difference in PR interval above 30 ms between the two modalities. In four of those cases, the P-wave was flat and difficult to discern.

Regarding the QRS duration, the smartwatch significantly underestimated the values compared to the 12-lead ECG (bias 18.4 ms (95% LoA -37.0 to + 73.8), *p* = 0.002; Fig. 3c). 28 participants (29%) had a difference > 30 ms between the two ECGs, mostly due to the watch underestimating the QRS interval through inaccurate determination of the S-wave’s ending (Fig. 2c).

The QT interval measured by the smartwatch had a tendency towards being underestimated (bias 28.8 ms (95% LoA -43.0 to + 100.5), *p* = 0.102; Fig. 3d). Fifty-one patients (60%) had a difference above 30 ms between the two readings. Outliers regarding the QT interval were usually due to difficulty deciding where the QRS complex began, and the T-wave ended (Fig. 2d). Furthermore, the smartwatch underestimated the QTc interval significantly (bias 30.6 ms (95% LoA − 54.8 to + 116.1), *p* < 0.001; Fig. 3e). 51 patients (60%) had a difference above 30 ms compared to the 12-lead ECG. The 51 outliers were not the exact same patients as for the QT interval despite the QTc interval being calculated from the QT.

### Interobserver variability

Interobserver agreement for the manual smartwatch measurements was excellent for all times and intervals (RR: ICC 1.00 (95% CI 0.99 to 1.00), *p* < 0.001; PR interval: ICC 0.97 (95% CI 0.90 to 0.99), *p* < 0.001); QRS interval: ICC 0.90 (95% CI 0.70 to 0.97), *p* < 0.001; QT interval: ICC 0.96 (95% CI 0.88 to 0.99), *p* < 0.001).

## Discussion

This study aimed to assess the accuracy of the Withings ScanWatch ECG´s ability to accurately determine ECG intervals in children and adolescents. The results showed excellent agreement between automated measurements of the standard 12-lead ECG and the smartwatch for HR, good agreement for the PR and QT interval and moderate agreement for the QRS and QTc interval. When using manual measurements of the smartwatch ECG tracings, agreement became good or excellent for all variables. Surprisingly, agreement between the two methods was weaker for the QTc interval than the QT interval.

To our knowledge, no studies have previously validated the ECG-intervals measured by a smartwatch in children or adolescents, but a few studies have been performed on adults using manual measurements of the Withings ScanWatch [13, 14]. Overall, these studies reported similar findings as ours. It is noteworthy to point out that these were studies on adult patients, that ECGs were not performed simultaneously, and – perhaps most importantly – Pearson’s correlation coefficient was evaluated rather than the ICC, which is a measure of agreement. With respect to PR intervals, the prior studies have identified a nonsignificant bias and a strong correlation for smartwatch ECGs with Pearson's correlation coefficients of 0.78 and 0.93, respectively [13, 14]. Our findings are consistent with that. Moving on to the QRS duration, previous studies have reported a Pearson's correlation coefficient between 0.80 and 0.97 (*p* < 0.005) [13]. In our data set we see moderate agreement when comparing automated smartwatch QRS duration to automated 12 lead ECG readings, which is increased when measuring the smartwatch derived QRS duration manually (ICC 0.7 vs 0.92). The agreement when comparing automated 12-lead and smartwatch ECGs was limited due to significant bias, i.e. the QRS interval was underestimated by the smartwatch. This has been described by others as well [13].

In our study, agreement between automated smartwatch measurements and automated 12-lead ECG for QTc was worse than for QT (ICC 0.53 vs 0.84, *p* < 0.001). This limited agreement in QTc was due to negative bias, which has been reported by others as well [13]. Thus, it seems like the automatically measured QTc interval may be underestimated by the smartwatch in children as well as adults.

The difference in agreement for QTc and QT comparing smartwatch and 12-lead ECG measurements became negligible when performing manual measurements on the smartwatch ECG (ICC 0.84 vs 0.95, *p* < 0.001). We observed that the smartwatch calculates QTc using average HR and QT interval from the entire 30 s reading, instead of the preceding RR interval for each beat to calculate the QTc. We conclude that manual determination of the QTc can optimize validity of the smartwatch based QTc, so that it may be tested further in clinical practice as a screening tool to detect changes in QTc over time.

For QRS duration and QTc, agreement of automated smartwatch with 12-lead ECG readings was limited (ICC 0.5–0.7,* p* < 0.001). A reason for this may be that the smartwatch only has one lead available (lead I). If due to the eletrical axis the amplitude is low in lead I, measurement inaccuracies may incur. Thus, if other leads were recorded by the smartwatch, this may result in more accurate measurements. This is a limitation when using a single lead ECG that may be overcome if the optimal lead for each patient is identified in the office setting first (i.e. the watch could be placed on the left foot to obtain lead II or III) [3].

The clinical relevance of our findings is that prolongation of the QT interval predisposes to life-threatening ventricular arrhythmias. Several common non-antiarrhythmic drugs cause QTc prolongation in adults and children, e.g. antihistamines, antipsychotics, antidepressants, or macrolides [2, 15]. Due to the rise in psychiatric diagnoses, some of these medicines have been used increasingly in children. Therefore, these children typically undergo ECG testing before and following start of QTc-prolonging medications and with changes in dosing. This practice is partly cumbersome for the patient and his/her family, but also uses medical resources to perform and interpret the ECG – which is particularly important as there is a wide-spread shortage of qualified medical staff. Therefore, replacing 12-lead ECGs done at the hospital or doctor’s office by regular home-based monitoring using a smartwatch may become an attractive alternative in the future – provided that the results are accurate. Our study showed that the accuracy of the smartwatch´s automated measurement of the QTc interval must improve before this can be made reality, or the interval must be manually measured by a cardiologist. In addition, the current study included only few patients with prolonged QTc. As QTc prolongation is associated with atypical T-wave morphology, further studies focusing on patients with known QTc-prolongation should be performed to determine diagnostic accuracy in this population.

### Study limitations

Limitations of this study are that manual measurements of shorter intervals have a limited temporal resolution. Thus, small measurement inaccuracies inherently result in larger relative inaccuracies, as we observed for the QRS duration. The paper speed was also not the same between the two ECGs (50 mm/s for the surface ECG, 25 mm/s for the smartwatch ECG), which may result in an inferior accuracy of the manual smartwatch ECG compared to the 12-lead ECG measurements, though the good overall interobserver variability suggests otherwise. Some measurements could not be obtained due to low amplitude of e.g. the P-wave.

## Conclusion

To our knowledge, this is the first study that validated smartwatch-derived ECG intervals in children. Automated smartwatch ECG intervals had excellent validity for HR. Validity for the PR interval, QRS duration and QT intervals was good and could be improved by performing manual measurements. The automated smartwatch QTc interval had the lowest level of agreement with 12-lead ECG measurements, but the validity can be improved by manually calculating the QTc interval. Currently, the automated QTc interval determined by smartwatch is not sufficiently precise to be used in clinical practice. We plan to conduct a longitudinal study in children who are started on QTc prolonging medications, in order to determine whether smartwatch ECGs can be used to monitor changes in QTc over time. In the future, automated QTc measurements using the smartwatch may become an alternative for patients at risk for QTc prolongation.

## Data Availability

Original data can be provided upon request.

## References

[1] Pasli S, Topcuoglu H, Yilmaz M, Yadigaroglu M, Imamoglu M, Karaca Y (2024) Diagnostic accuracy of apple watch ECG outputs in identifying dysrhythmias: a comparison with 12-Lead ECG in emergency department. Am J Emerg Med 79:25–32 38330880 10.1016/j.ajem.2024.01.046

[2] McNally P, McNicholas F, Oslizlok P (2007) The QT interval and psychotropic medications in children: recommendations for clinicians. Eur Child Adolesc Psychiatry 16(1):33–47 16944043 10.1007/s00787-006-0573-0

[3] van der Zande J, Strik M, Dubois R, Ploux S, Alrub SA, Caillol T et al (2023) Using a smartwatch to record precordial electrocardiograms: a validation study. Sensors (Basel) 23(5):2555 10.3390/s23052555 PMC10007514 36904759

[4] Spaccarotella CAM, Polimeni A, Migliarino S, Principe E, Curcio A, Mongiardo A et al (2020) Multichannel electrocardiograms obtained by a smartwatch for the diagnosis of ST-segment changes. JAMA Cardiol 5(10):1176–1180 32865545 10.1001/jamacardio.2020.3994 PMC7466842

[5] Maille B, Wilkin M, Million M, Rességuier N, Franceschi F, Koutbi-Franceschi L et al (2021) Smartwatch electrocardiogram and artificial intelligence for assessing cardiac-rhythm safety of drug therapy in the COVID-19 pandemic. The QT-logs study. Int J Cardiol 331:333–9 33524462 10.1016/j.ijcard.2021.01.002 PMC7845555

[6] Leroux J, Strik M, Ramirez FD, Racine HP, Ploux S, Sacristan B et al (2023) Feasibility and diagnostic value of recording smartwatch electrocardiograms in neonates and children. J Pediatr 253:40–5.e1 36113637 10.1016/j.jpeds.2022.09.010

[7] Littell L, Roelle L, Dalal A, Van Hare GF, Orr WB, Miller N et al (2022) Assessment of Apple Watch Series 6 pulse oximetry and electrocardiograms in a pediatric population. PLOS Digit Health 1(8):e0000051 36812630 10.1371/journal.pdig.0000051 PMC9931318

[8] Gropler MRF, Dalal AS, Van Hare GF, Silva JNA (2018) Can smartphone wireless ECGs be used to accurately assess ECG intervals in pediatrics? A comparison of mobile health monitoring to standard 12-lead ECG. PLoS ONE 13(9):e0204403 30260996 10.1371/journal.pone.0204403 PMC6160047

[9] Roguin A (2011) Henry Cuthbert Bazett (1885–1950)–the man behind the QT interval correction formula. Pacing Clin Electrophysiol 34(3):384–388 21091739 10.1111/j.1540-8159.2010.02973.x

[10] Harris PA, Taylor R, Thielke R, Payne J, Gonzalez N, Conde JG (2009) Research electronic data capture (REDCap)–a metadata-driven methodology and workflow process for providing translational research informatics support. J Biomed Inform 42(2):377–381 18929686 10.1016/j.jbi.2008.08.010 PMC2700030

[11] Harris PA, Taylor R, Minor BL, Elliott V, Fernandez M, O’Neal L et al (2019) The REDCap consortium: building an international community of software platform partners. J Biomed Inform 95:103208 31078660 10.1016/j.jbi.2019.103208 PMC7254481

[12] Koo TK, Li MY (2016) A guideline of selecting and reporting intraclass correlation coefficients for reliability research. J Chiropr Med 15(2):155–163 27330520 10.1016/j.jcm.2016.02.012 PMC4913118

[13] Pengel LKD, Robbers-Visser D, Groenink M, Winter MM, Schuuring MJ, Bouma BJ et al (2023) A comparison of ECG-based home monitoring devices in adults with CHD. Cardiol Young 33(7):1129–1135 35844104 10.1017/S1047951122002244

[14] Touiti S, Medarhri I, Marzouki K, Ngote N, Tazi-Mezalek A (2023) Feasibility and reliability of whintings scanwatch to record 4-lead electrocardiogram: a comparative analysis with a standard ECG. Heliyon 9(10):e20593 37842608 10.1016/j.heliyon.2023.e20593 PMC10568083

[15] De Ponti F, Poluzzi E, Cavalli A, Recanatini M, Montanaro N (2002) Safety of non-antiarrhythmic drugs that prolong the QT interval or induce torsade de pointes: an overview. Drug Saf 25(4):263–286 11994029 10.2165/00002018-200225040-00004

